# Environmental impacts from the widespread implementation of ocean thermal energy conversion

**DOI:** 10.1007/s10584-025-03944-1

**Published:** 2025-05-10

**Authors:** Anna G. Nickoloff, Sophia T. Olim, Michael Eby, Andrew J. Weaver

**Affiliations:** School of Earth and Ocean Sciences, University of Victoria, PO Box 1700, Victoria, BC V8 W 2Y2 Canada

**Keywords:** Ocean Thermal Energy Conversion (OTEC), Renewable energy systems, Earth system modelling, Climate change mitigation, Environmental impact assessment

## Abstract

Ocean thermal energy conversion (OTEC) is a renewable energy system that could potentially displace significant amounts of fossil fuel-generated electricity. This study presents numerous multi-century simulations of the University of Victoria Earth System Climate Model, a coupled climate-carbon cycle model, to better understand the global-scale environmental impacts of the widespread implementation of OTEC at varying total power levels (3, 5, 7, 10, and 15 TW). Environmental impacts include reduced warming of the sea surface by up to 3.1 ºC, increased heat uptake at intermediate depths, and enhanced biological production compared to a fossil fuel intensive control scenario. At year 2100, OTEC-induced mixing contributes roughly 60% of the relative cooling, while the remainder is from OTEC-related emission reductions. Once OTEC is terminated, all relative cooling is caused by accumulated emissions reductions. If acting alone, the residual effect of OTEC-induced mixing would contribute to a minor relative warming of the sea surface. The effect of OTEC on the expansion of known oxygen minimum zones was minimal. In many circumstances, OTEC deployment opposes the projected impacts of climate change. Relative to a high carbon emissions control scenario, OTEC deployment is associated with less surface warming, a smaller increase in surface water pCO_2_, a suppression of ocean acidification, and significantly smaller declines in the strength of the Atlantic Meridional Overturning Circulation. Despite the potential engineering challenges and economic costs, early indications suggest that the large-scale implementation of OTEC could make a substantial contribution to climate change mitigation.

## Introduction

Since the Industrial Revolution, energy use and associated CO_2_ emissions have vastly increased due to rapid population growth, increased urbanization, accelerated production, and technological advances. Atmospheric CO_2_ concentrations have increased from a preindustrial level of about 280 ppm to 415 ppm by the year 2020, resulting in a global temperature increase of approximately 1.2 ºC (Chen et al. 2021). The WHO has described the direct and indirect effects of climate change as the current greatest threat to human health (World Health Organization 2021). Despite their well-documented negative environmental impacts, fossil fuels provide most of the world’s energy (Curtin et al. 2019; IEA 2023). A major adoption of renewable energy systems is required to generate the rapid greenhouse gas emissions reductions needed to mitigate anthropogenic climate change while still supporting humanity’s increasing energy needs.

Ocean Thermal Energy Conversion (OTEC) generates electricity from the thermal gradient between warmer surface water and cooler deep ocean water (DOW). OTEC plants utilize warm water from the sea surface and cool DOW pumped from the ocean’s interior, typically from around 1 km depth. Locations with temperature gradients suitable for OTEC are constrained to deep, warm seas in the tropics. There has been much research and debate regarding the extent to which OTEC could contribute to the world’s energy needs and the technology’s environmental safety and economic viability.

The transport of large volumes of cool, nutrient-rich DOW to the euphotic zone is inherent to OTEC. The introduction of DOW to surface waters could have wide-reaching influences on the physiochemical properties and thermal structure of the ocean and potentially severe impacts. Currently, much of the research surrounding the environmental impacts of OTEC deployment has focused on local biological ramifications (U.S. Department of Energy 1979; Coastal Response Research Center 2012; Devault and Péné-Annette 2017; Aresti et al. 2023). While this analysis is vital, the global environmental impacts of OTEC must also be considered. The thermal structure of the ocean influences a myriad of oceanographic properties (e.g., rates of ice-sheet melt, ENSO variability, the strength of the ocean circulation, and concentrations of dissolved gases in seawater). While climate change is driving the need for innovation in climate engineering and carbon emission reductions, it also necessitates careful consideration and a full understanding of the potential environmental impacts of proposed solutions.

### Previous OTEC modelling studies

Modelling attempts have focused primarily on either quantifying the size of the OTEC reservoir (Nihous 2005, 2007a, 2007b; Rajagopalan and Nihous 2013a, 2013b, 2013c) or the modelling of site-specific OTEC deployment (VanZwieten et al. 2017; Devault and Péné-Annette 2017; Langer et al. 2022). Less research has looked at the impact of widespread OTEC deployment on ocean systems despite the clear need for this analysis.

In several studies (Nihous 2005, 2007a, 2007b), modelling of a one-dimensional oceanic water column was conducted to investigate the effects of OTEC on the thermal structure of the ocean. These studies found OTEC to be associated with a cooling of the mixed layer and warming at depth. While one-dimensional analyses provide some insight into the effects of OTEC, three-dimensional modelling is required to understand OTEC’s influence on ocean circulation and other regional impacts.

The first three-dimensional study to quantify the effects of OTEC using an oceanic general circulation model was by Rajagopalan and Nihous (2013a). They used the Massachusetts Institute of Technology General Circulation Model (MITgcm) ocean model with a resolution of 4° × 4° but without atmospheric, sea ice and carbon-cycle components. Their main conclusions were that OTEC would result in an augmentation of the oceanic thermohaline circulation, and cooling of the tropics would be balanced by warming at higher latitudes. A study with a higher-resolution (1° × 1°) version of the same ocean model broadly confirmed these findings (Rajagopalan and Nihous 2013b).

The effectiveness and ramifications of artificial ocean upwelling, among other forms of climate engineering, were the subject of a 2014 study using the UVic ESCM (Keller et al. 2014). Artificial upwelling was modelled to transport water from 1000 m depth to the surface. While the total amount of upwelling is similar to some of the OTEC scenarios in this study (about 20 Sv), the area over which upwelling occurred was much larger than the area suitable for OTEC. Another key distinction is that no power is being produced, and with no associated reduction in emissions, changes to the ocean reflect increased upwelling rates alone. The study found that artificial upwelling is initially effective at carbon storage, but its effectiveness decreases on longer timescales. Artificial upwelling was also linked to increases in pCO2 and decreases in pH due to the upwelling of carbon-rich deep waters. Notably, it was also found that while artificial upwelling was associated with cooling the sea surface while operational, once this upwelling was stopped, temperatures rose to values greater than a fossil fuel-intensive scenario without climate engineering initiatives.

In 2015, Kwiatkowski et al. used a complex three-dimensional ocean–atmosphere coupled model, the Community Earth Systems Model (CESM), to explore the oceanic and atmospheric response to increased vertical transport in the ocean (Kwiatkowski et al. 2015). The study showed that increased vertical mixing was associated with initial atmospheric cooling relative to control values. However, after 50 years of simulation, atmospheric temperatures increased above those of the control simulation. The magnitude of the increase in vertical transport is stronger than what might be expected with OTEC and applied globally. However, this study could be viewed as a crude proxy of the effects of OTEC-induced mixing, particularly in the absence of other fully coupled model representations.

Jia et al. (2018) furthered previous investigations into the effect of OTEC on ocean systems with the MITgcm model by including a simple atmospheric feedback. The study confirmed much of the findings of Rajagopalan and Nihous’ previous papers. The paper also concluded that OTEC-induced mixing reinforced the Atlantic Meridional Overturning Circulation (AMOC) and could induce an AMOC-like feature in the North Pacific Ocean.

Using the same model configuration as presented in this study, Nickoloff et al. (2025) used the UVic ESCM to investigate the amount of extractable energy and the climate mitigation potential of OTEC. It was found that between 2030 and 2500, OTEC power production between 3 and 15 TW resulted in cumulative emission reductions of 323 to 981 Pg of carbon by 2500 relative to a fossil fuel-intensive control scenario without OTEC deployment. The combination of the substantial OTEC-related carbon emissions reductions and enhanced mixing of cool deep water to the sea surface led to globally averaged atmospheric temperature decreases of 1.0 ºC to 4.0 ºC relative to control values by year 2500, depending on the level of OTEC power generation.

While others have described the localized environmental impacts of OTEC, this study further investigates the global-scale impacts associated with OTEC using a fully coupled climate-carbon cycle model. It is the first study to demonstrate how these environmental impacts may vary with climate change and the amount of OTEC energy extracted. The study also utilizes a novel sub-grid parameterization of OTEC mixed effluent outflow to better represent vertical plume dynamics and an adaptive OTEC plant deployment scheme that reduces the number of plants (and thus cost) required for a given level of OTEC energy production. This analysis is also the first to include modelled emissions reductions associated with OTEC deployment and to demonstrate the interconnected environmental impacts of OTEC-related enhanced mixing and carbon emissions reductions. This study has a unique focus on how climate change with varying levels of OTEC energy extraction would impact sea-surface temperatures (with implications for climate variability), dissolved-oxygen levels, carbonate chemistry, nutrient concentrations, biological production and changes to the large-scale ocean circulation. These experiments ultimately provide a more comprehensive view of the potential global environmental impacts of OTEC.

## Methods

### Model description

The UVic ESCM was employed to produce several multi-century simulations. The UVic ESCM is a fully coupled global model of intermediate complexity with zonal and meridional grid resolutions of 3.6º and 1.8º, respectively. The model comprises a vertically integrated two-dimensional energy-moisture balance atmospheric model (Weaver et al. 2001), a dynamic-thermodynamic sea ice model (Hibler 1979; Hunke and Dukowicz 1997; Bitz and Lipscomb 1999), a primitive equation oceanic general circulation model (Pacanowski 1995), a comprehensive carbon cycle model, and a land surface scheme with a dynamic global vegetation model (Cox et al. 2000).

In this study, OTEC plants are presumed to be offshore autonomous platforms and are not constrained to be near land. Warm water is drawn from the sea surface and mixed with cold water pumped from around 1,100 m depth through a large diameter pipe. Mixed effluent is released at the specified discharge depth (about 20 m). Entrainment is parameterized over a circular area (500 m radius) and discretized as ten vertical columns. Each column has a surface area defined by ten concentric thick-walled vertical cylinders each with a wall thickness of 50 m. These concentric cylinders extend from the discharge point to the edge of entrainment. See Fig. 1 in Nickoloff et al. (2025). At each model timestep, the initial depth profile for each column is prescribed to be equivalent to that of the course resolution model and the volume of effluent is distributed linearly from a maximum value at the centre of discharge to zero at the furthest extent of entrainment. This allows discharge per unit area to decrease with distance from the point of discharge. The innermost cylinder represents the portion of the plume with the least entrainment and the deepest vertical penetration of effluent. The outermost cylinder represents the portion of the plume with the most entrainment and the lowest amount of penetration. Only changes in the vertical structure of the water column affect the large-scale model. Therefore, this parameterization of entrainment is not intended to simulate the horizontal distribution of the effluent plume, but rather to capture the decreased vertical mixing with increased entrainment. Vertical advection and convection are calculated for the ten columns separately, and these columns are then mixed laterally with the larger ocean grid cell at all depths. This lateral mixing is weighted by the surface area of columns relative to the surface area of the rest of the ocean model grid cell. See Nickoloff et al. (2025) for additional model details.

**Fig. 1:**
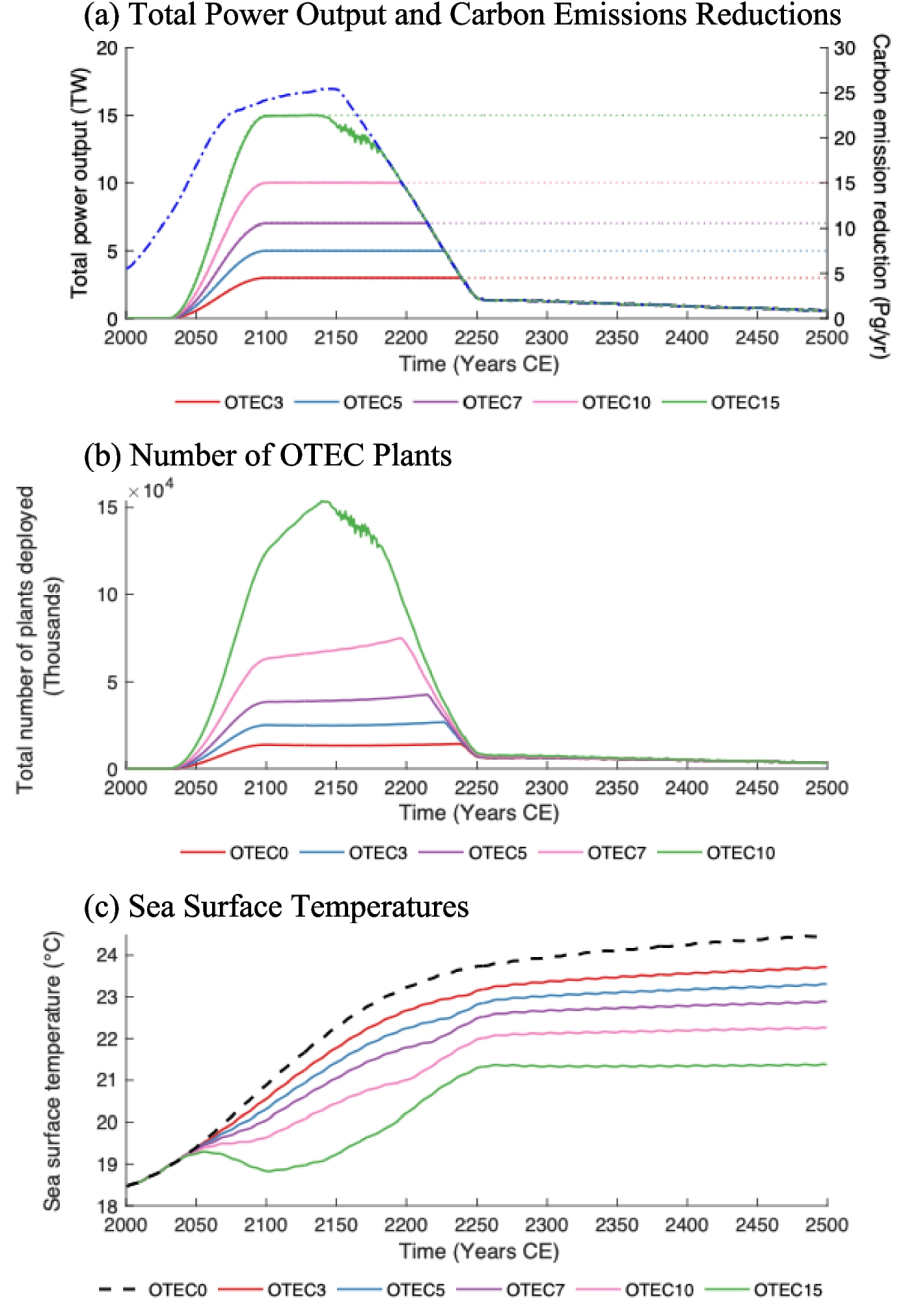
(**a**) Total OTEC power output in TW (terawatts) on the left axis and OTEC-associated carbon emission reductions in Pg/yr of carbon on the right axis. The solid lines denote the net power output from OTEC (left axis) and the corresponding emissions reductions (right axis) for each simulation. The dotted lines mark the power goals, and the dashed blue line represents the climate mitigation power demand limitation. (**b**) Total number of OTEC plants deployed. (**c**) Globally averaged SSTs in ºC. The dashed black line represents *OTEC0* and in all figures, the red, blue, violet, pink, and green solid lines denote the *OTEC3*, *OTEC5*, *OTEC7*, *OTEC10*, and *OTEC15* scenarios, respectively

The power produced by an OTEC plant is approximated by Eq. 1 (see Rajagopalan & Nihous 2013b for more details):

1 $${P}_{net}={\omega }_{cw} \rho {c}_{p} {\varepsilon }_{tg} \left(\frac{9}{80}\frac{\Delta {T}^{2}}{T}-\frac{9}{200}\right)$$

Here ${\omega }_{cw}$ represents the volume flow rate of OTEC deep seawater (m^3 ^s^−1^), $\rho$ is the mean seawater density (1,025 kg m^−3^), ${c}_{p}$ is the specific heat (4,000 J kg^−1^ K^−1^), ${\varepsilon }_{tg}$ is the turbo-generator efficiency (0.75), $T$ is the intake seawater temperature, and $\Delta T$ is the temperature difference between surface and deep seawater intakes. The numerical coefficients account for a flow rate ratio of 1.5 of surface-to-deep seawater and seawater pumping power losses equal to 30% of the turbo-generator output at standard conditions.

For an average OTEC plant to produce about 100 MW in “average” tropical water column temperatures, a volume flow rate of 314 m^3 s−1^ is specified for each plant. This flow rate could be generated with a 10 m diameter pipe and a water velocity of 4 m s^−1^. These parameters are somewhat arbitrary and may not reflect plant configurations in future OTEC deployments but are used as a rough estimate of potential configurations and are consistent with previous studies (Rajagopalan and Nihous 2013c; Jia et al. 2018).

OTEC plants are relocated if OTEC power generation falls below a set minimum power. OTEC plants are decommissioned and not relocated in three circumstances: (1) OTEC power production meets the set power goal, (2) climate mitigation power demands are satisfied, or (3) there are no remaining locations suitable for OTEC. OTEC power production goals ramp up to reach specified peak power production following a raised negative cosine function. The climate mitigation power demand parameter limits OTEC power production to the power that would have otherwise been sourced from fossil fuels, given a specific emissions scenario. It is assumed that OTEC power production would result in an emissions reduction of 1.5 Pg of carbon per year for every 1 TW of electricity produced. This ratio is roughly based on current estimates of the carbon intensity of all electrical power production (Moore et al. 2022) and assumes that only 60% of electricity is derived from fossil fuels (Ritchie et al. 2020). The calculated emission reduction is subtracted from the emissions specified in a future climate change scenario. Suitable OTEC locations are grid cells with sufficient space and thermal gradients for OTEC power production. The set minimum area of 200 square kilometres per plant would result in a minimum average distance between plants of about 16 km.

The required number of OTEC plants is derived from the specified OTEC power goal (which varies with time as production increases) and the annual average power that plants can produce in a given grid cell (Eq. 1). Power generation becomes less efficient over time as thermal gradients become depleted, and additional plants are required to reach the power production goal. Newly built or relocated OTEC plants are placed into locations with the greatest annual average power generation potential. Only one plant is placed in each suitable grid cell at a time to avoid rapid depletion of thermal gradients. If the number of plants to be placed is greater than the number of grid cells that meet the minimum power and area requirements, deployment is started again with the grid cell with the largest potential power production. This is continued until all available plants are placed or there are no areas left that meet the minimum power and area requirements. In the latter case, surplus plants are added to the total available for future placement. See Nickoloff et al. (2025) for additional model details.

### Experimental design

All simulations used initial conditions derived from a long 10,000-year equilibrium model spin-up at the year 850 with only seasonally varying forcing. The spin-up was integrated to the year 2000 with transient historical forcings specified by the Climate Model Intercomparison Project Five (Eby et al. 2009). This formed the initial condition for all subsequent experiments. From the years 2000 to 2300, extended Representative Concentration Pathway (RCP) 8.5 forcings were specified. Beyond the year 2300, year 2300 forcing was specified with only seasonal variations (Zickfeld et al. 2013). Using specified RCP8.5 CO_2_ concentrations, the UVic ESCM was employed without OTEC deployment to diagnose CO_2_ emissions from the year 2000 to the end of the modelled period in the year 2500. These emissions were specified in subsequent experiments, which allowed the model to react to emission reductions from OTEC power production rather than be constrained by the CO_2_ concentrations specified by the RCP8.5 scenario. RCP8.5 was selected as the most appropriate pathway as, unlike the other pathways (2.6, 4.5, and 6.0), RCP8.5 accounts for neither the employment of renewable technologies nor strategies for reducing greenhouse gas emissions. Diagnosed emissions from RCP8.5 are solely used in these experiments as it is essential to use an emission scenario that does not already account for the type of emission reductions attributed to OTEC.

Several scenarios with varied OTEC power goals were generated, beginning in the year 2000 and terminating in the year 2500. In each experiment, OTEC power generation commenced in the year 2030 and increased smoothly following a raised negative cosine function to reach the desired OTEC power production by the year 2100. Experiments were produced for OTEC power rates of 3, 5, 7, 10, and 15 TW, referred to as *OTEC3, OTEC5, OTEC7, OTEC10*, and *OTEC15*, respectively. OTEC power production rates range from the more conservative estimates of OTEC power limits to more optimistic ones while remaining within previous estimates for OTEC power production as limited by environmental safety (Avery and Wu 1994). Additionally, a control simulation (*OTEC0*) was generated in which global warming occurs under RCP8.5 conditions without any OTEC power generation. Model simulations were also conducted where OTEC power generation began in the year 2030, reached peak power generation by year 2100, and abruptly stopped at the year 2100. These modelled scenarios allow for the analysis of temperature conditions post-OTEC power generation. This is discussed further in Sect. 3.2.2. Modelled scenarios were also generated for each level of OTEC power generation where the CO_2_ emission reductions associated with OTEC are excluded. These simulations reflect deployment where OTEC replaces non-emitting forms of energy and allow for the contributions of emission reductions and OTEC-induced mixing to be examined.

## Results and discussion

### OTEC carbon emissions reductions and climate change mitigation

After the commencement of OTEC in the year 2030, power generation in *OTEC3, OTEC5, OTEC7, OTEC10,* and *OTEC15* increases steadily to reach specified maximum power levels by the year 2100. Following this, power production rates are held constant until generation becomes limited by the climate mitigation power demand or a lack of suitable locations for OTEC. All simulations sustain the specified level of OTEC power generation for the first 200 to 250 years of the modelled period. Thereafter, power generation become limited by climate mitigation power demand or lack of suitable plant locations (OTEC15) and diminishes to less than 1.5 TW for the remainder of the simulation. OTEC scenarios with greater power output become limited by climate mitigation power demand before those with lower output. By year 2500, all OTEC scenarios produce a mean power of 0.6 TW.

By the year 2500, power generation causes a 323 Pg (*OTEC3*) to 981 Pg (*OTEC15*) decrease in cumulative carbon emissions relative to *OTEC0*. These reductions represent 36% to 111% of the year 2023 cumulative anthropogenic carbon emissions since 1750 (Ritchie et al. 2020). Without OTEC deployment (*OTEC0*), global average atmospheric CO_2_ concentrations reach the year 2500 values of 1930 ppm, whereas simulations with significant deployment (*OTEC15*) experience year 2500 values of 623 ppm. The decreased emissions, coupled with the upwelling of cool DOW to the sea surface, result in globally averaged atmosphere surface temperature reductions of 1.0 to 4.0 ºC relative to control values. In the first 100 years of the modelled period, while OTEC is operating at a high level, OTEC-induced mixing contributes roughly 60% of the observed cooling, while the remainder is the result of OTEC-related emission reductions. Once OTEC power production diminishes, all cooling results from the sustained effect of emissions reductions, as past OTEC-induced mixing alone would result in a small net warming effect.

### Ocean temperature changes

Global average sea surface temperatures (SST) rise continuously across all modelled simulations. The increase is greatest in the *OTEC0* and decreases incrementally as the level of OTEC power generation increases (Fig. 1). By the year 2500, global mean SST reductions range from 0.8 ºC (*OTEC3*) to over 3 ºC cooler (*OTEC15*) than *OTEC0.* In the first century of OTEC power generation, SST is primarily controlled by OTEC-induced mixing. In the year 2100, OTEC-induced mixing contributes 63% of the observed sea surface cooling, and the remaining 37% is a product of emissions reductions. As time progresses, OTEC emissions reductions accumulate in the atmosphere, and their effect on SSTs strengthens. During this same period, OTEC power generation rates decrease, and OTEC-related emissions reductions become the dominant SST control. By the year 2245, the entire cooling signal is caused by emissions reductions, and OTEC-induced mixing contributes to a net warming of SSTs. This is covered further in Sect. 3.2.1.

Relative to *OTEC0*, OTEC-related cooling is experienced globally but concentrated in regions where OTEC is operational, particularly in the West Pacific. Much of the relative temperature decrease is sustained after most OTEC is terminated (Fig. 1). By the year 2500, globally averaged surface waters still experience net temperature increases relative to the year 2000 as the high CO_2_ concentrations present in RCP8.5 cause warming that overwhelms OTEC-induced cooling. Simulations with power generation rates above 7 TW experience regional cooling below year 2000 values for around 100 years while OTEC is operating at a high level. At the highest level of OTEC power generation (*OTEC15*), waters in the West Pacific warm pool experience maximum temperature decreases of 3.3 ºC, relative to the year 2000.

**Fig. 2:**
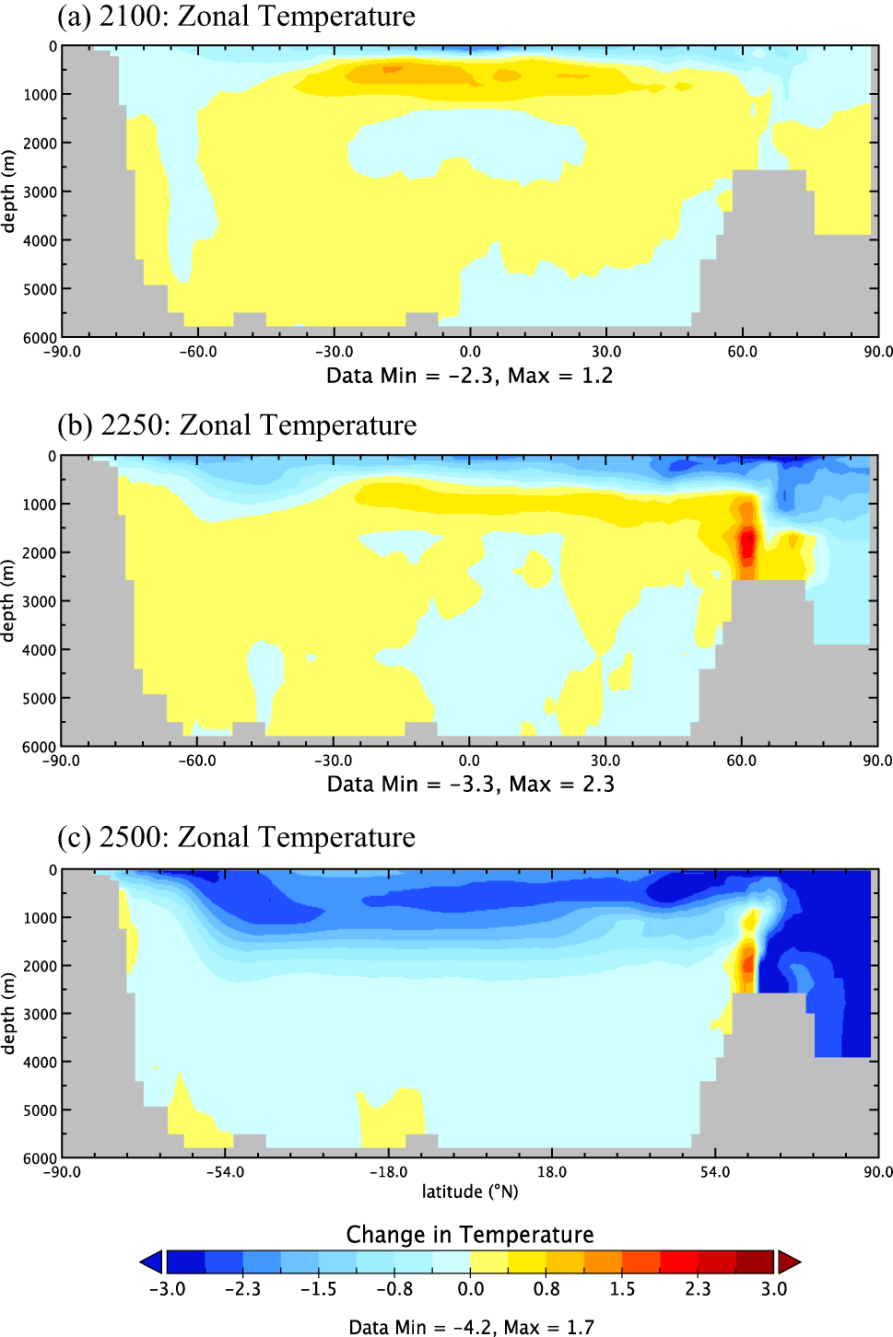
Change in global zonally averaged ocean temperature in ºC for the 10 TW OTEC (*OTEC10*) RCP8.5 scenario in 2100 (**a**), 2250 (**b**), and 2500 (**c**) relative to the same year in an RCP8.5 scenario without OTEC (*OTEC0*)

The relative surface temperature decreases are balanced by some heating in the ocean interior. In the year 2100, much of the ocean experiences temperature increases at depth. Peak warming is centred at 18 ºS at depths of 500—1000 m (Fig. 2a). The warming is concentrated in the equatorial Pacific between the warm pool and the Americas. At depths below 1000 m, changes to ocean temperatures are relatively small and mainly stay within half a degree of zero. The magnitude of this warming is augmented with increasing OTEC power generation, but the spatial and temporal trends remain consistent. OTEC operations involve the removal of cool DOW at depth, which gets replaced by relatively warmer, mixed water, resulting in the observed temperature increase in the ocean interior. Additionally, relative cooling at the surface enhances ocean heat uptake. When this heat gets circulated to the ocean's interior, it further contributes to temperature increases.

#### Potential warming in areas without OTEC

By year 2250, the relative warming at depth primarily occurs around 60 ºN from depths of 1000 to 2500 m (Fig. 2b), and this likely relates to the reinforcement of the AMOC and a shift in the area of deep-water formation (discussed further in Sect. 3.6). Relative surface water cooling extends from 40 ºN to 85 ºN and reaches depths around 1000 m. While surface waters experience a cooling relative to *OTEC0*, absolute SSTs rise above 2000 levels due to the dominant warming signal from anthropogenic climate change. Ocean temperatures will also be affected by the prevention of some sea ice melt in OTEC scenarios. By the year 2500, nearly the entire water column experienced cooling relative to *OTEC0* aside from a few regions at depth in the Southern Ocean, and the warming cell described above (Fig. 2c). Strong relative cooling occurs in both Antarctic Intermediate Waters and Arctic Bottom Water.

**Fig. 3:**
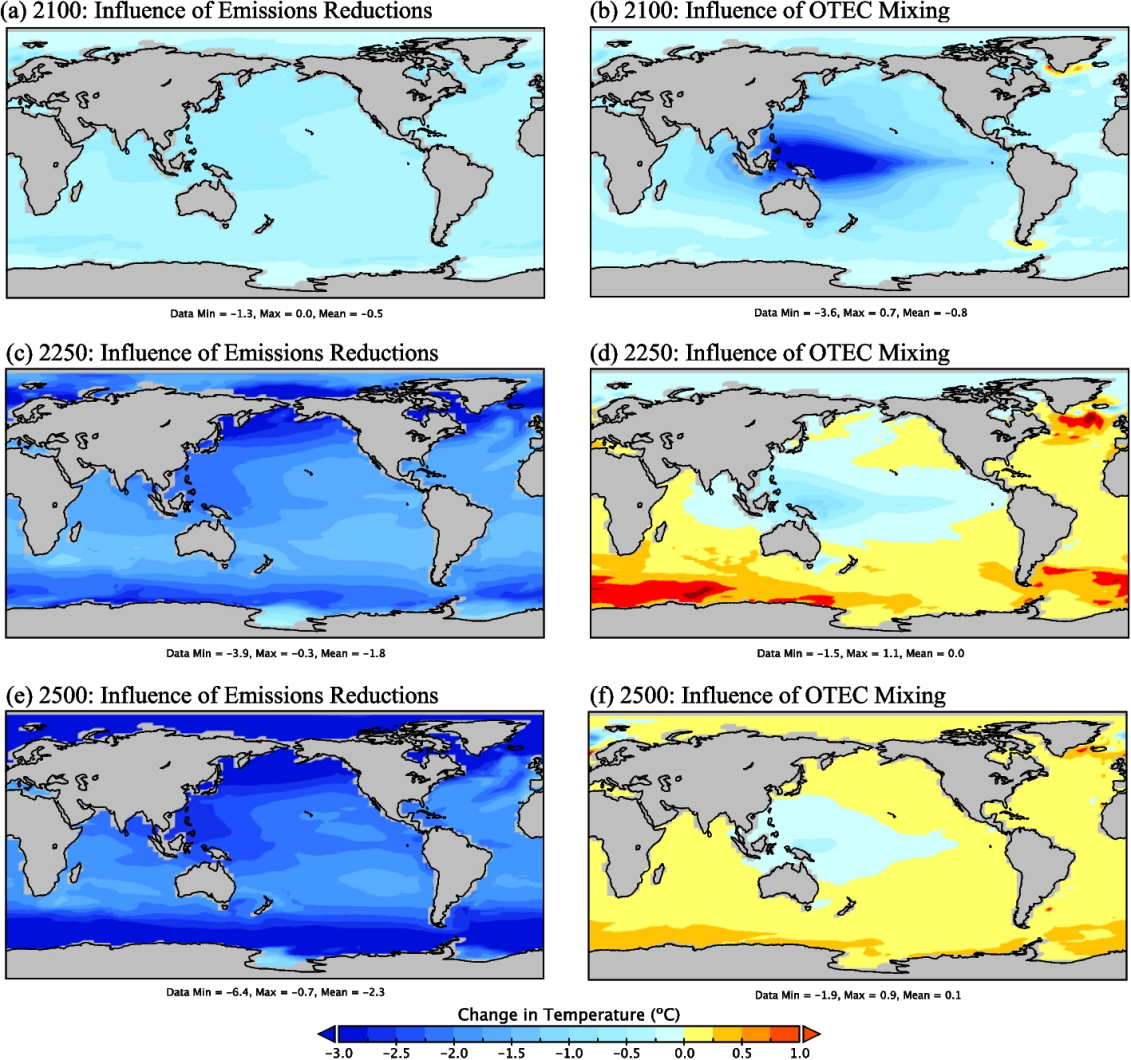
Change in SSTs in ºC. The left panels show the influence of OTEC-related emissions reductions (*OTEC10* with emissions reductions minus *OTEC10* without emissions reductions), and the right panel shows the influence of OTEC-induced mixing (*OTEC10* without emissions reductions minus *OTEC0*) in years 2100 (**a** and **b**), 2250 (**c** and **d**), and 2500 (**e** and **f**). Warm tones (yellow/orange) denote areas that have warmed, and cool tones (blue) indicate areas that have cooled

It has been proposed that the relative cooling seen in areas of OTEC power generation would be balanced by relative heating at higher latitudes (Rajagopalan and Nihous 2013a, 2013c; Jia et al. 2018). This was not the case when the effect of emission reductions and OTEC-induced mixing were considered. By 2500, global mean SSTs range from 0.8 ºC (*OTEC3*) to 3.1 ºC (*OTEC15)* below *OTEC0* values. OTEC-related emission reductions cause ubiquitous cooling of the surface oceans relative to the *OTEC0* throughout the modelled period, with a local maximum of 6.4 ºC below *OTEC0* in polar regions by the year 2500 (Fig. 3a, c, e).

When only considering the impact of OTEC-induced mixing, some relative warming is seen in regions without OTEC deployment (Fig. 3b, d, f), particularly after OTEC is largely terminated, as was found in previous studies (Rajagopalan and Nihous 2013a, 2013c; Jia et al. 2018). During periods of significant OTEC power generation (years 2030–2150), SSTs are less than *OTEC0* values aside from small areas around Greenland and the Drake Passage, which rise by less than a degree (Fig. 3b). While relative cooling remains present in areas of OTEC deployment once OTEC power generation has effectively ceased (beyond year 2200), regions without such deployment experience warming above *OTEC0* values (Fig. 3d, f). By the year 2500, the warming is greatest in the Atlantic and the Southern Ocean, where maximum SSTs rise from 0.7 ºC (*OTEC3*) to 1.0 ºC (*OTEC15*) above the *OTEC0* scenario. If OTEC mixing is considered in isolation, this relative warming could have wide-reaching effects on rates of ice-sheet destabilization of the Greenland and Antarctic Ice Sheets and patterns of DOW formation in the North Atlantic.

**Fig. 4:**
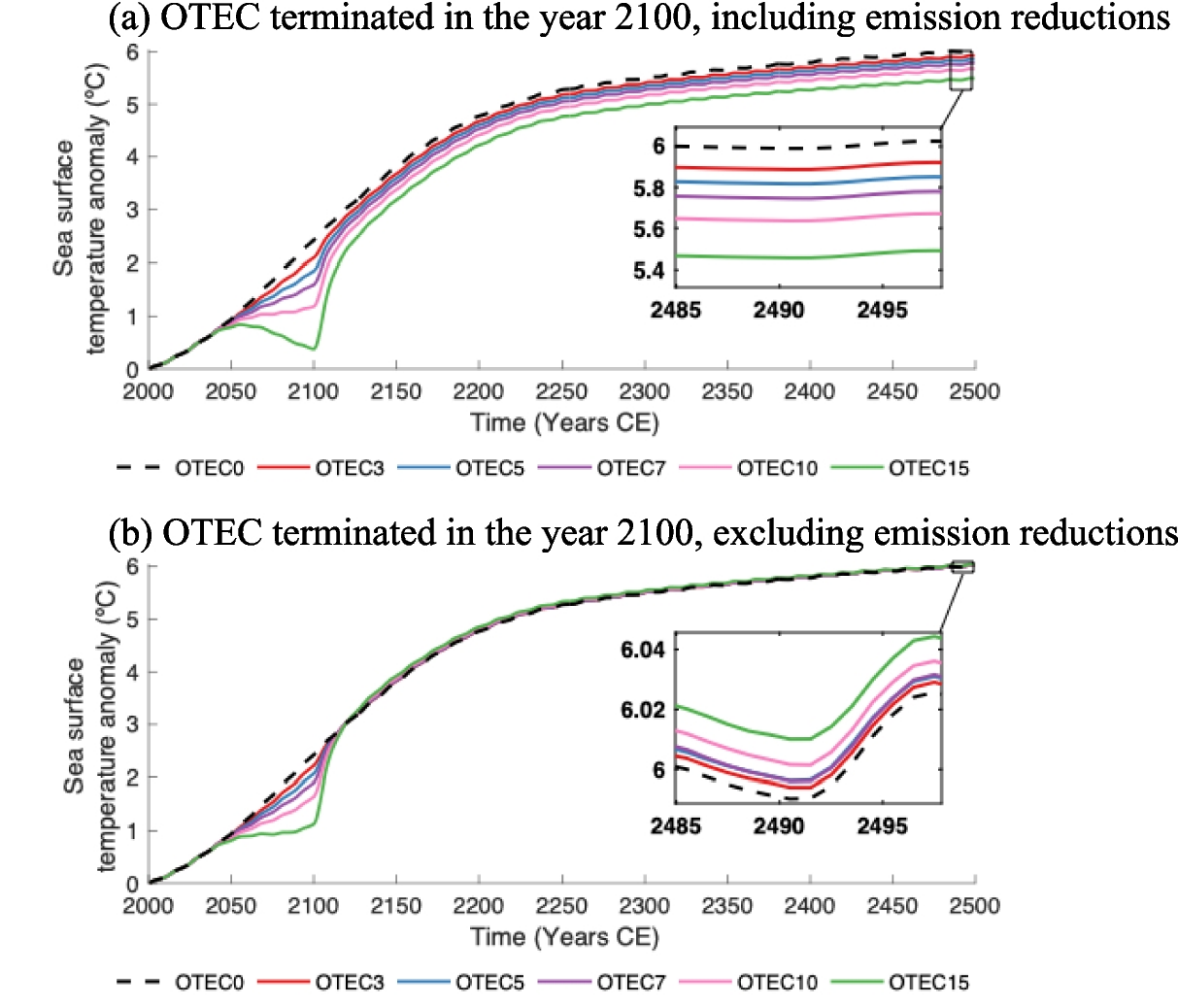
Globally averaged sea surface temperatures in ºC including (**a**) and excluding (**b**) OTEC-related emission reductions in *OTEC0* (dashed black), *OTEC3* (red), *OTEC5* (blue), *OTEC*7 (violet), *OTEC10* (pink), and *OTEC15* (green). In these simulations, all OTEC plants were rapidly shut down in the year 2100

To investigate the reaction of ocean temperatures to the termination of OTEC, model simulations were produced where OTEC power generation was completely discontinued by the end of the year 2100. In these circumstances, SSTs remain lower relative to the *OTEC0* values even after power generation is halted, provided that OTEC-related emission reductions are considered (Fig. 4a). The magnitude of the relative decrease is related to the level of OTEC power generation, and this remains consistent after the termination of OTEC. Even with OTEC only producing power between the years 2030 and 2100, the emissions reduction is substantial enough in all OTEC scenarios that global average SSTs remain suppressed even 400 years after power generation is ceased (Fig. 4a).

This is not the case when OTEC-related emission reductions are excluded (Fig. 4b). SSTs under these conditions rise rapidly after the suspension of OTEC power generation in the year 2100 and reach values slightly above those of the *OTEC0.* During periods of OTEC deployment, temperature changes are concentrated in the equatorial West Pacific with maximum year-2100 temperature decreases of 1.4 ºC (*OTEC3*) to 5.0 ºC (*OTEC15*) below *OTEC0* values. Once OTEC is terminated, SSTs rebound slightly above *OTEC0* values with a global mean increase of up to 0.02 ºC above *OTEC0* values by the year 2500 (*OTEC15*). Temperature changes primarily occur in deep water formation regions. By the year 2500, local SST maxima of 0.1 ºC (*OTEC0*) to 0.2 ºC (*OTEC15*) above *OTEC0* occur in polar regions, particularly near Greenland. In the Norwegian Sea, SSTs experienced a local minimum of 0.04 ºC (*OTEC0*) to 0.7 ºC (*OTEC15*) below *OTEC0*.

A similar phenomenon occurs in the OTEC simulations that do not terminate in the year 2100 but become limited by climate mitigation power demands around the year 2250. When only OTEC-induced mixing is considered, surface waters initially experience a relative cooling but rebound slightly above *OTEC0* values once OTEC power generation is diminished. By year 2500, global mean SSTs range from 0.004 ºC (*OTEC0*) to 0.1 ºC (*OTEC15*) above *OTEC0* values. Local maximum SSTs range from 0.7 ºC (*OTEC3*) to 1.0 ºC (*OTEC15*) above *OTEC0*. While these temperature rebounds are greater than those that occur when OTEC is terminated in the year 2100, both temperature rebounds are relatively minor. OTEC termination does not appear to be linked to dramatic temperature rebounds above *OTEC0,* which would further contribute to the increased oceanic warming associated with climate change. However, potential localized rebounds could still be significant as they are concentrated in areas near large ice sheets (as seen in Fig. 3b) and may be harmful to biological communities in affected areas.

#### Alteration of west pacific gradient

**Fig. 5:**
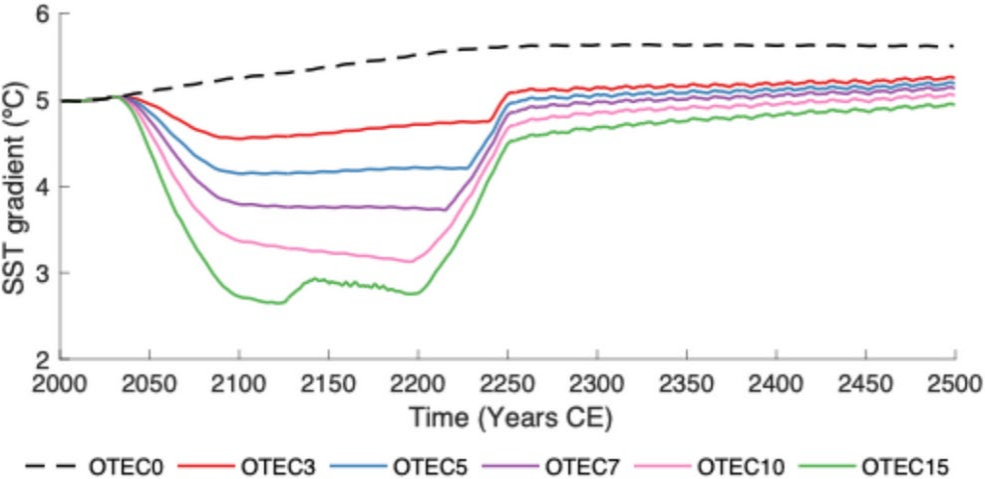
The difference in sea surface temperatures between 2.5°N-S, 117–173°E and 2.5°N-S, 205–275°E in ºC (WPG) from the year 2000 to 2500 for the *OTEC0* (dashed black), 3 TW (red), 5 TW (blue), 7 TW (violet), 10 TW (pink), 15 TW (green) scenarios

OTEC plant placement is concentrated in the equatorial West Pacific to maximize power production. OTEC plants are associated with decreased SSTs in the Western Pacific warm pool and a weakening of the West Pacific Gradient (WPG) over the first two centuries when OTEC is operational (Fig. 5). Following Coats and Karnauskas (2017), the WPG is defined as the difference in SSTs between 2.5°N-S, 117–173°E and 2.5°N-S, 205–275°E. The El Niño-Southern Oscillation (ENSO) is driven, at least in part, by the thermal contrast between these western and eastern sections of the Pacific (Amaya 2019; Zinke et al. 2021). The weakening of the WPG may affect the ENSO frequency and strength. As surface air temperatures rise due to climate change, it remains unclear how the WPG will be affected (Lee et al. 2022). The WPG in *OTEC0* increases over the modelled period by over 0.5 ºC, and this finding is supported by historical data, which shows the WPG strengthened from the years 1880 to 2005 (Lee et al. 2022).

Relative to *OTEC0*, the WPG is appreciably reduced in scenarios with OTEC power generation (Fig. 5). OTEC-induced mixing occurs predominantly in the West Pacific. Therefore, SSTs in this region are reduced more than those in the Eastern Pacific. Once the majority of OTEC power generation has ceased (by year 2250), the WPG in all OTEC scenarios increases rapidly and remains elevated (Fig. 5). In all OTEC scenarios, the resulting year 2500 SST gradients are altered less from year 2000 levels than the gradient in the *OTEC0* scenario. While it is unclear exactly how these changes may affect the ENSO, shifting ENSO patterns and intensities of any kind may disrupt wind and rainfall patterns across the tropics and have a plethora of global side effects. The response of ENSO under such a shift should be investigated further with an Earth System Model with a more complex atmospheric component.

### Dissolved oxygen concentrations

In the last 50 years, oxygen levels at intermediate depths in the equatorial Pacific and Atlantic have experienced an annual decrease of 0.34 µmol/kg (Stramma et al. 2008). This decline is predicted to intensify with increased rates of warming (Stramma et al. 2008). Due to global warming, absolute values of surface water oxygen decrease in all modelled scenarios, although the magnitude of the decrease is reduced with increasing OTEC power generation. The suppression of oxygen decline in surface waters is initially driven predominantly by OTEC-induced mixing. In the year 2100, 62% of the changes to global average surface oxygen concentrations can be attributed to enhanced mixing, and 38% are from emissions reductions. By year 2250, sustained OTEC-related emissions reductions become the dominant driver of reduced decline in surface water oxygen for the remainder of the modelled period.

Relative to *OTEC0,* the implementation of OTEC is associated with higher oxygen concentrations in the uppermost 600 m of the water column and lower between 600 and 1900 m depth. This suggests that OTEC deployment may lessen the effect of shallow oxygen minimum zones and worsen those of deeper origin. While these changes in oxygen are consistent with changes in ocean temperature alone, increased productivity and changes in ocean circulation also play a role (Tiano et al. 2014). Oxygen minimum zones commonly occur between depths of 100 and 1500 m, and the two major oxygen minimum zones in the Pacific are in the east, away from the concentrated OTEC deployment in the Western Pacific Warm Pool (Kamykowski and Zentara 1990; Paulmier and Ruiz-Pino 2009). Preliminary results suggest it is unlikely that OTEC would play a significant direct role in the expansion or reduction of oxygen minimum zones.

### Carbonate chemistry

Over the modelled period, there is an increase in the pCO_2_ of surface waters in all scenarios. The increase is greatest in the *OTEC0* scenario and is reduced in the scenarios with OTEC implementation. As with oxygen, surface water pCO_2_ is influenced by the changes in SSTs, OTEC-induced mixing rates, and the stimulation of primary productivity. Unlike oxygen, the greatest driver of changes in surface ocean pCO_2_ is increasing atmospheric CO_2_. In the year 2100, OTEC-related emissions reductions contribute 88% to the suppression of surface pCO_2_ increase, while any changes due to enhanced mixing contribute 12%. By the year 2300, OTEC-induced mixing contributes less than 1%, and this signal is driven almost entirely by emissions reductions. Global average surface water pH decreases over the modelled period in all simulations. OTEC scenarios experience relatively lower surface water pCO_2_ and diminished alteration of pH values. By the year 2500, *OTEC0* has average surface water pH values of 7.4, whereas the scenarios with OTEC have average pH values ranging from 7.5 (*OTEC3*) to 7.9 (*OTEC15)*. This reduction of ocean acidification could be important for ecosystems vulnerable to lower pH, particularly calcifying organisms like coral reefs and many types of plankton. Likewise, global average sea surface alkalinity experiences smaller decreases due to OTEC deployment relative to *OTEC0*. This relative increase in surface alkalinity is influenced by relatively higher pH, elevated photosynthetic rates, and increased mixing of high alkalinity DOW. Alkalinity is vital in buffering changes to ocean pH. Smaller reductions in surface alkalinity may benefit ocean ecosystems, mitigate ocean acidification, and enhance the uptake of atmospheric CO_2_ (Middelburg et al. 2020).

### Nutrient concentrations and biological productivity

The mixing of large volumes of nutrient-rich deep waters augments nutrient concentrations in surface waters. The volume flow rate of DOW, and therefore the magnitude of nutrient concentration changes, is proportional to the level of OTEC power generation. Global average surface phosphate and nitrate concentrations experience sharp increases following the start of OTEC deployment in the year 2030. Phosphate concentrations remain elevated over the modelled period, while nitrate concentrations experience a decrease between years 2100 and 2200 before rising again. The persistence of phosphate and drawdown nitrate indicates that nitrate is the more limiting nutrient in most tropical regions (Geider and La Roche 2002; Van Mooy et al. 2009; Moore et al. 2013). Both nutrient depth profiles exhibit the typical pattern of a biologically active tracer– depleted in surface waters and enriched in deep water. With OTEC deployment, nutrient concentrations near the ocean surface increase, at intermediate depths (500–3000 m), decrease and at greater depths (> 3000 m), again increase. Surface water concentrations increase as nutrient-rich waters are upwelled to the ocean surface. At intermediate depths, these nutrient-rich waters are being replaced with nutrient-poor waters. With the increase in near-surface nutrients, primary productivity and detritus increase and as these materials degrade, nitrate and phosphate concentrations increase at depth.

Most of the world’s oceans are limited by either the availability of macronutrients like nitrogen (N), phosphorus (P) or silicon (Si), or essential micro-nutrients like iron (Fe) or nickel (Ni) (Arrigo et al. 1979; Sakka Hlaili et al. 2006; Leinen 2008; Pan et al. 2016) and the introduction of macronutrients from depth enhances biological production in surface waters. By the year 2100, global mean ocean net primary productivity experiences a 0.06 (*OTEC3*) to 0.14 (*OTEC15*) nmol m^−3^ s^−1^ of N increase, with regional maximum increases of 1.7 (*OTEC3*) to 3.0 (*OTEC15*) nmol m^−3^ s^−1^ of N in areas where OTEC-induced mixing is occurring.

### Changes to large-scale ocean circulation

**Fig. 6:**
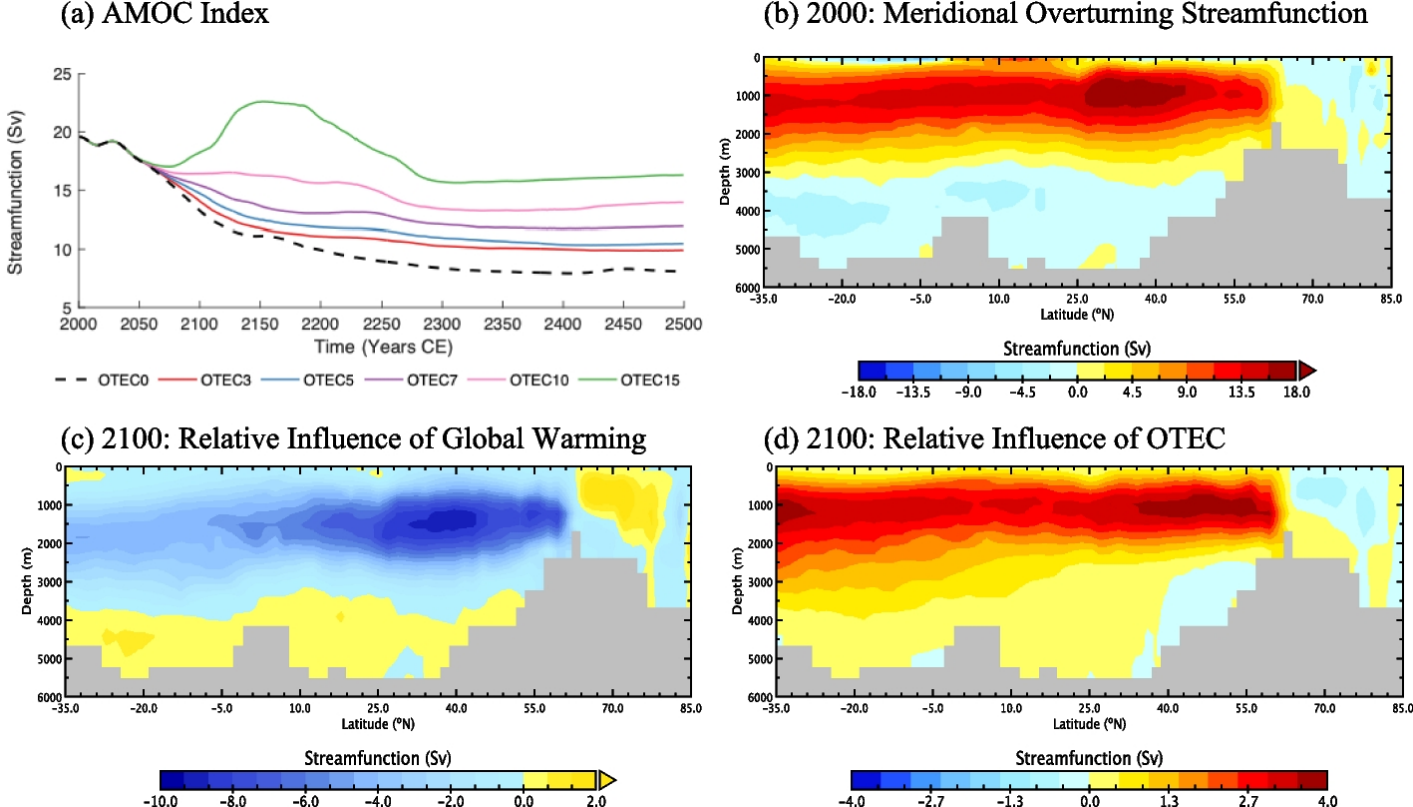
(**a**) Maximum North Atlantic meridional overturning streamfunction index in Sverdrups (1 Sv = 10^6^ m^3^ s^−1^) from 2000 to 2500 in *OTEC0* (dashed black), *OTEC3* (red), *OTEC5* (blue), *OTEC7* (violet), *OTEC10* (pink), and *OTEC15* (green), (**b**) Zonally averaged meridional overturning streamfunction in *OTEC0* in 2000, (**c**) Streamfunction anomaly in 2100 relative to 2000 for *OTEC0,* (**d**) Streamfunction anomaly for *OTEC10* relative to *OTEC0* in 2100, in the Atlantic Ocean, in Sverdrup (Sv). Warmer colours indicate clockwise flow, and cooler colours, counterclockwise flow

While uncertainty remains (Lobelle et al. 2020), it is generally accepted that global warming will cause at least a temporary weakening of the AMOC (Cheng et al. 2013; Nobre et al. 2023; Madan et al. 2024) while OTEC is projected to strengthen it (Rajagopalan and Nihous 2013a, 2013c; Jia et al. 2018; Rau and Baird 2018). While all simulations experience a net decrease in overturning strength by year 2500, all OTEC power extraction scenarios result in a stronger AMOC by the year 2500 relative to *OTEC0* (Fig. 6a). Between years 2000 and 2500, the AMOC index in *OTEC0* falls by 11.5 Sv, whereas *OTEC15* shows a reduction of only 3.3 Sv (Fig. 6a). The AMOC index is defined here to be the maximum positive Atlantic streamfunction value between 25 and 65 ºN and between 500 and 2000 m depth. At the peak of OTEC power generation, *OTEC15* briefly experiences AMOC indices above year 2000 levels with a maximum value of 22.6 Sv (Fig. 6a). For the first 150 years of the simulation, changes in AMOC indices are primarily influenced by OTEC-induced mixing. OTEC-induced mixing contributes 89% of the change in the year 2100, while the emission reduction contributes 11%. Between years 2150 and 2300, OTEC-induced mixing and OTEC emission reductions contribute to the change in circulation strength in roughly equal proportions. After the year 2300, OTEC operates at low levels, and the reduction in cumulative emissions becomes the primary influence on changes to the AMOC index. By the year 2500, 84% of the increase in circulation is from emissions reductions, while the remaining 16% is due to OTEC-induced mixing.

The relative increase in AMOC strength has been attributed to significantly increased rates of.

diapycnal mixing brought on by large-scale OTEC power generation (Rajagopalan and Nihous 2013c; Rau and Baird 2018). The coupled heat injection to DOW and cooling of surface waters decreases the stratification (density stability) of the water column and leads to increased vertical mixing rates. Numerical modelling has shown that the strength of the overturning circulation relies in part on the global, background diapycnal diffusion coefficient (Zhang et al. 1999). OTEC-related vertical mixing similarly decreases stratification, making it easier for water, forced by deep water formed in polar regions, to move toward the surface.

Changes to the AMOC are the combined effects of both global warming and the implementation of OTEC. To examine the effects of both driving forces, the initial North Atlantic meridional overturning streamfuction, as well as streamfunction anomalies from *OTEC0* in the year 2100 relative to the year 2000 (the effect of global warming) and *OTEC10* in the year 2100 relative to *OTEC0* in the year 2100 (the effect of OTEC) are presented in Fig. 6b, c and d, respectively. Modelled values show appreciable reductions in deep water formation in the North Atlantic between the years 2000 and 2100 in *OTEC0* from 60 ºN to 35 ºS at depths above 3000 m (Fig. 6c). These reductions are concentrated in a recirculation cell that extends from the equator to around 50 ºN with maximum relative values of −9.33 Sv and correspond to a slowing of the AMOC. OTEC deployment is associated with a relative increase in the strength of the overturning circulation, partially counteracting the decrease from warming, with a maximum relative value of −3.9 Sv (Fig. 6d).

## Conclusions

OTEC provides a unique opportunity for relatively clean, continuous energy. While modelled scenarios suggest OTEC could generate immense amounts of power, this would not be without environmental concerns. While it may be possible for OTEC to displace a considerable amount of fossil fuel-generated power, the advantages of OTEC are lessened if it replaces other forms of zero-emissions energy. Despite the engineering challenges, economic costs, and potential ecological disruptions during its operational phase, OTEC is a significant contender for use in climate change mitigation.

While OTEC is largely operational, OTEC-related emissions reductions initially make a small but escalating contribution to the suppression of surface water warming, reduction of AMOC strength decline, curtailment of ocean acidification, and the limitation of shallow oxygen minimum zones. OTEC-induced mixing initially exerts a more considerable influence, aiding in reducing SSTs, stabilizing the AMOC, and stimulating net primary productivity. In the year 2100, roughly 60% of the observed global cooling is from OTEC-induced mixing, while the remainder is from OTEC-related emission reductions. The mixing of cool DOW to the sea surface also increases oceanic uptake of CO_2_ and further supports the reduction of shallow oxygen minimum zones. However, while OTEC-induced upwelling cools the tropical sea surface, it would alter local ecosystems by introducing cold, acidic deep water to the sea surface, and it may even result in minor, localized polar warming. The severity of these environmental concerns scales with the level of power generation. Of the five power scenarios considered in detail, the greatest alteration of ocean systems occurs in the 15 TW scenario, while the 3 TW scenario indicates relatively minor impacts.

After OTEC is no longer operational, residual effects of OTEC-induced mixing are associated with lasting alteration of oceanic physiochemical properties. If OTEC power production reduces carbon emissions by displacing fossil-fuel-generated power, the relative surface air and sea cooling is sustained and experienced globally. However, if OTEC only displaces other forms of zero-emission energy, and there are no OTEC-related emission reductions, cooling in regions with OTEC could be balanced by warming elsewhere. OTEC-induced mixing would then contribute to global warming slightly above the RCP8.5 control after OTEC termination. While this warming is relatively small, SST increases are concentrated in polar regions, which may affect rates of deep water formation and contribute to ice sheet destabilization.

Emission reductions facilitated by OTEC offer substantial and permanent benefits, aiding in sustained temperature mitigation, AMOC preservation, and the suppression of ocean acidification and oxygen minimum zones. OTEC-induced mixing without emission reductions demonstrates fewer long-term benefits, with its primary impact being a delay in warming, albeit resulting in minor extra surface warming over centuries. In many ways, the environmental impacts of OTEC oppose those of climate change, although some of these benefits would also come from adopting other renewable energy systems. By the year 2500, producing between 3 and 15 TW of power with OTEC would result in cumulative emission reductions equivalent to 36% to 111% of historical carbon emissions from years 1750 to 2023, relative to an RCP 8.5 control scenario without OTEC deployment. Surface air temperatures are lower by 1 ºC to 4 ºC by the year 2500 relative to the scenario without OTEC. Even without OTEC’s considerable potential for emissions reductions, OTEC mixing could delay some aspects of climate change until other mitigation efforts can be developed.

There remain several areas where further research is required. Firstly, it is not possible to comment on the acute effects of OTEC on ocean biota. A more complex biological model is needed to investigate how OTEC deployment will impact different ecosystems and animal species. Chemical and physical changes associated with OTEC will vary regionally and have composite effects on biota. Additional studies are needed to assess the ramifications of OTEC-induced changes to ocean circulation on modes of climate variability, like ENSO or monsoons, as well as ice sheet stability, large-scale nutrient distributions, and biological production. Further potential environmental costs of OTEC, such as alterations of wind patterns, extreme weather events, rainfall patterns, and cloud cover, remain unexplored and warrant attention in future analysis with a more intricate atmospheric model. However, there are also potential environmental benefits of OTEC that require further attention. OTEC could reduce storm severity by suppressing SSTs in known tropical cyclone generation zones, stabilizing or reducing ENSO variability and strength, and limiting coral bleaching.

OTEC offers a promising pathway for continuous, renewable energy generation while contributing to emissions reductions and temporary climate mitigation. However, its deployment introduces environmental trade-offs, including potential localized ecosystem disruptions and polar warming, with impacts scaling with power generation levels. While OTEC's benefits, particularly in reducing fossil fuel emissions, are substantial, further research is needed to fully understand its effects on ocean biota, circulation patterns, and broader climatic systems before large-scale implementation can be pursued.

## Data Availability

The code and data used in this study are available from the authors on request.
